# The impact of maximized resection and standardized systemic therapy on overall survival in adult patients with thalamic gliomas

**DOI:** 10.3389/fonc.2025.1681695

**Published:** 2025-10-30

**Authors:** Junjie Wang, Xiaodong Niu, Tao Chang, Yuxin Quan, Lloyd Mulenga Mwibwe, Yanhui Liu, Xiang Wang, Yuan Yang, Qing Mao

**Affiliations:** Department of Neurosurgery, West China Hospital, Sichuan University, Chengdu, Sichuan, China

**Keywords:** thalamus, glioma, oncology, system therapy, prognosis

## Abstract

**Objective:**

This study aims to explore the impact of maximized resection and standardized systemic surgery + chemoradiotherapy(SRC) on the survival prognosis of adult thalamic glioma, and to construct a clinical prognosis model of adult thalamic glioma.

**Methods:**

A retrospective analysis was conducted on adult cases of thalamic glioma who underwent craniotomy in the Department of Neurosurgery of West China Hospital of Sichuan University from 01/03/2009 to 01/03/2024. Firstly, Kaplan-Meier survival analysis and subgroup analysis were conducted. Secondly, COX regression and LASSO-COX regression were performed on 12 variables respectively to screen the variables and construct a prognostic model. Then, the efficacy of different models was compared to select the optimal model to construct a prognostic nomogram for the overall survival of thalamic glioma.

**Results:**

Total of 192 adult patients with thalamic glioma were included in this study, of whom 84 underwent surgery only, 41 underwent surgery + radiotherapy/chemotherapy(SR/SC), and 67 completed SRC. Among them, 79 patients(41.1%) completed gross-total tumor resection during the operation, and 113 patients(58.9%) completed non-gross-total tumor resection. The efficacy of the three models was compared. The optimal LASSO-COX model included five variables that affected the overall survival(OS) of thalamic glioma (EOR, diagnosis, preoperative hydrocephalus, postoperative KPS, treatment). Then, these five variables were utilized to develop prognostic nomograms for predicting the 6-, 12-, 24-, 36-, and 60-month OS. The nomogram shows good predictive ability and clinical practicability. Finally, the risk stratification system based on the prognostic nomogram effectively divided patients into the high-risk group and the low-risk group.

**Conclusions:**

Maximized tumor resection within safe parameters with standardized systemic SRC significantly prolong the OS of patients with thalamic glioma. The survival prognosis nomogram based on LASSO-COX regression in this study can be used as a practical tool for predicting the survival probability of patients with thalamic glioma.

## Introduction

Thalamic gliomas are a subset of primary brain tumors originating in the dorsal thalamus, frequently extending into critical functional regions such as the brainstem, internal capsule, and basal ganglia (1, 2). These tumors represent approximately 1-5% of all intracranial neoplasms (3, 4). Historically, the challenges associated with surgical intervention for thalamic gliomas have been profound due to the deep-seated location of the thalamus, its proximity to essential brain structures and vascular networks, and the infiltrative nature of these tumors (5, 6). These challenges typically result in low total resection rates, high postoperative complication and mortality rates, and generally poor prognoses, thus leading to a primary focus on biopsy and postoperative adjuvant therapies (7–11).

Since the 1980s, advances in medical imaging technologies such as CT and MRI, along with the development of surgical aids like microscopes, have fostered the evolution of minimally invasive neurosurgery. These innovations have significantly enhanced the extent of thalamic glioma resections and have markedly reduced perioperative mortality and morbidity, achieving a mortality rate of less than 1% (12–17). Recent reports from neurosurgical centers globally, including our prior studies, underscore that maximizing tumor resection within safe parameters can extend overall survival in patients with thalamic gliomas, thereby supporting the role of surgical intervention in their treatment (12, 15, 18, 19).

Despite these advancements, thalamic gliomas still yield less favorable outcomes compared to gliomas in the cerebral hemispheres, in terms of surgical resection extent, postoperative Karnofsky Performance Status (KPS) scores, and overall survival (20, 21). Currently, a comprehensive study evaluating the benefit of standardized systemic therapy in adult thalamic gliomas is lacking. The advantage of employing standardized therapies—such as surgery combined with radiotherapy and chemotherapy—effective for hemispheric gliomas, remains contentious when applied to the distinct anatomical context of thalamic gliomas. In our study, we retrospectively analyzed prognostic data from 192 patients with thalamic gliomas, systematically evaluating whether systemic therapy confers greater benefits compared to surgery alone. We also investigated the influence of various molecular pathological characteristics on systemic therapy outcomes, aiming to provide more refined guidance for the treatment of thalamic gliomas in future clinical practice.

## Materials and methods

### Data collection and study population

**Figure 1:**
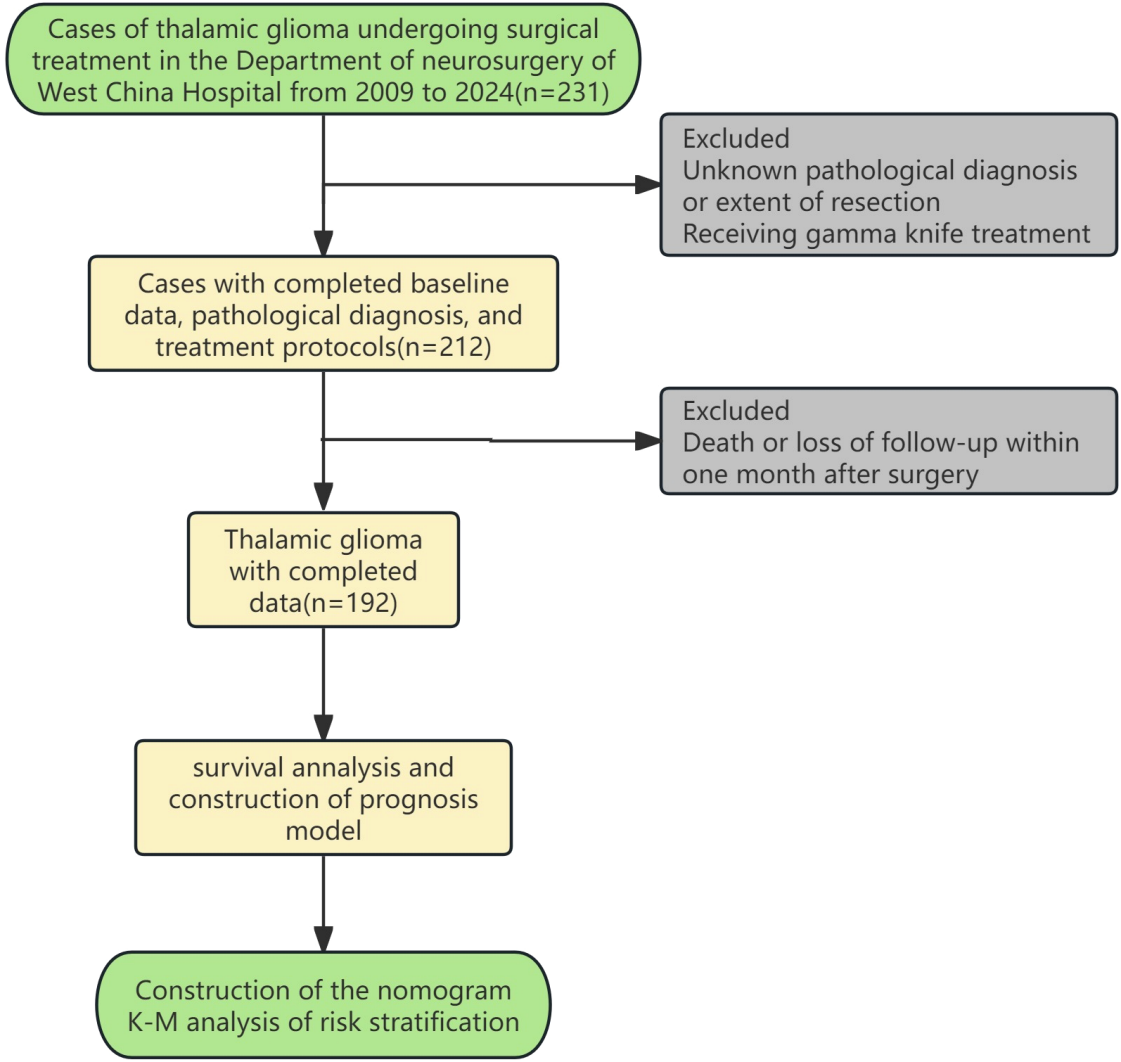
The flow diagram of case inclusion and exclusion.

Medical records of patients who underwent craniotomy to remove thalamic space occupying lesions at the Department of Neurosurgery, West China Hospital, Sichuan University from 01/03/2009 to 01/03/2024 and were pathologically confirmed as glioma were reviewed and collected. Patients lacking critical information such as age, gender, pathological diagnosis, treatment protocol (surgery only, surgery and postoperative radiotherapy/chemotherapy, surgery and postoperative chemoradiotherapy), and OS and outcome were excluded. Patients who died within 1 month after surgery were excluded. The remaining patients were divided into surgery group, surgery and postoperative radiotherapy/chemotherapy group, and surgery and postoperative chemoradiotherapy group according to the treatment regimen they received(considering that the number of cases of postoperative radiotherapy and postoperative chemotherapy is relatively small, the two are combined into one group). The specific inclusion and exclusion process is shown in **Figure 1**. This study has been approved by the Ethics Review Committee of West China Hospital of Sichuan University, before accessing the data, all data had been completely anonymized, authors had no access to information that could identify individual participants during or after data collection, and the ethics committee waived the informed consent requirement.

### Statistical analysis

Patient characteristics and covariates included age, gender, tumor characteristics (pathological diagnosis, molecular characteristics), degree of resection (gross total resection, non-gross total resection), treatment plan (surgery, SR/SC, SRC), preoperative and postoperative hydrocephalus, preoperative and postoperative KPS scores, OS (months), and survival status. Pathological diagnoses include anaplastic astrocytoma(AA), diffuse astrocytoma(DA), diffuse midline glioma(DMG), glioblastoma(GBM), and others. The molecular characteristics of tumor included IDH mutation, H3K27M mutation and chromosome 1p/19q co-deletion. GTR was determined based on a combination of intraoperative assessment by the surgical team and postoperative MRI evaluation indicating no apparent abnormal tumor tissue. The postoperative KPS score was assessed consistently at 2 weeks after surgery. The primary outcome of this study was OS. OS was defined as the time interval between diagnosis and last follow-up or death from any cause. Survival status was recorded at the last follow-up or confirmation of patient death.

The Chi-square test and Wilcoxon rank sum test were used to compare demographic, clinical, and pathological features among patients in the surgery, SR/SC, and SRC cohorts. Continuous variables, including age, preoperative and postoperative KPS scores, were calculated and grouped by R software package. Different covariates were divided into different subgroups. Kaplan-Meier survival curves were drawn, and Log-rank test was used to compare the differences in OS among the surgery cohort, the SR/SC cohort, and the SRC cohort within each subgroup. For pairwise comparisons among all treatment groups, we used Bonferroni correction to adjust for P values. In order to retain the statistical information to the greatest extent, this study standardized the continuous variables(age, preoperative KPS and postoperative KPS), then LASSO-COX regression analysis was used for variable screening and to establish a prognostic model. LASSO regression is a statistical method used for the construction of linear regression models and feature screening. It is particularly suitable for medium and small samples, can automatically screen important variables, handle multicollinearity of variables, reduce the risk of overfitting, and improve the generalization ability of the model. lambda.1se is the optimal lambda value, which can maximize the simplification of the Model and is used to construct the LASSO-COX model (Model 1) (λ is determined through 10-fold cross-validation). Considering that the degree of tumor resection can reduce the tumor burden of patients to varying degrees, affect the subsequent therapeutic effect and prognosis, based on the principle of mandatory retention of prior knowledge, we added EOR on the basis of Model 1 to construct a new LASSO-COX Model (Model 2). The prediction efficiencies of the two models for OS were compared through the Harrel consistency index (C-index), Akaike Information Criterion (AIC), and Bayesian Information Criterion (BIC). The time-dependent ROC curves of the two models were plotted and the corresponding area under the curve(AUC) values were calculated to compare the discriminatory ability of the two models for survival outcomes at different time points. The clinical application value of the two models was compared through decision curve analysis(DCA). Then, the optimal model is selected as the final prediction model. Multivariate COX regression analysis was conducted to calculate the hazard ratios and 95% confidence intervals of each variable, and the results were used to draw forest plots.

Then, internal Validation is carried out through 200 times of Bootstrap Validation. This method can maximize the utilization of all samples for modeling and performance evaluation, and correct the performance index (C-index) of the original model to obtain the true predictive ability of the model and test its stability. Calibration curves were used to evaluate the accuracy of the model’s predictive ability. The final selected variables were used to construct a prognostic nomogram to predict the survival probabilities of thalamic glioma at 6-, 12-, 24-, 36-, and 60 months. Risk stratification was performed according to the nomogram, and relevant K-M curves were drawn.

In addition to the primary multivariable Cox proportional hazards model, we performed supplementary analyses on three molecular pathological markers—H3K27M mutation, IDH mutation, and 1p/19q codeletion—to comprehensively assess their prognostic value. First, univariable Cox regression analyses were conducted to evaluate the independent prognostic significance of each marker. Subsequently, we performed statistical power analyses to evaluate the ability of the current sample size to detect the effects of these molecular alterations. Power calculations were based on the observed effect sizes and event distributions. For 1p/19q codeletion, due to the absence of event occurrences in the positive group, the required sample size for detecting such an effect was estimated based on an assumed HR of 0.3.

A two-sided P value <0.05 was considered to indicate statistical significance. Statistical analysis were performed using R (version 4.4.1) software.

## Results

### Patient clinical, pathological and demographic characteristics

Total of 192 patients with thalamic glioma were included in this study, of whom 84 underwent surgery only, 41 underwent SR/SC, and 67 completed SRC. The median age of the patients was 39.5 years old, and the range was 64 years old. There were 80 female patients, accounting for 41.7% of the total sample, and 58.3% of the male patients. The median preoperative KPS score was 80 and the median postoperative KPS score was 50. 29.7% of the patients had hydrocephalus before surgery, 11.7% of the patients had hydrocephalus after the surgery. There were statistically significant differences among the three groups in the two indicators of preoperative hydrocephalus and postoperative KPS score. The median postoperative KPS score was 50, and there was a statistical difference between the three groups, which may be because patients with low postoperative KPS score could not tolerate postoperative chemoradiotherapy. The presence of hydrocephalus before the operation may indicate a large tumor volume and an early expansive growth, causing obstruction of the ventricular circulatory system, often suggesting a low-grade glioma. This might be the reason for the differences among the three groups. The mean OS of 192 patients was 14.0 months, among which the mean total OS of patients who only underwent surgery was 7.94 months, the mean OS of patients who underwent surgery plus radiotherapy/chemotherapy was 14.6 months, and the mean OS of patients who completed surgery plus chemoradiotherapy was 21.1 months, with statistically significant differences among the three groups. The OS of thalamic glioma patients can be significantly prolonged by systematic and standardized treatment. Detailed clinical, pathological and demographic characteristics are shown in **Table 1**.

**Table 1:** Demographic, clinical, and pathological characteristics associated with treatment plan in thalamus glioma.

Variables	Surgery	SR/SC	SRC	Overall	P.value
	(N=84)	(N=41)	(N=67)	(N=192)	
Age						
Mean (SD)	41.5 (14.7)	37.9 (16.2)	40.6 (15.0)	40.4 (15.1)	0.59
Median [Min, Max]	42.0 [18.0, 73.0]	36.0 [14.0, 70.0]	40.0 [15.0, 78.0]	39.5 [14.0, 78.0]	
Gender						
Female	38 (45.2%)	18 (43.9%)	24 (35.8%)	80 (41.7%)	0.69
Male	46 (54.8%)	23 (56.1%)	43 (64.2%)	112 (58.3%)	
Extent of resection						
GTR	39 (46.4%)	11 (26.8%)	29 (43.3%)	79 (41.1%)	0.207
nGTR	45 (53.6%)	30 (73.2%)	38 (56.7%)	113 (58.9%)	
Diagnosis						
AA	9 (10.7%)	5 (12.2%)	11 (16.4%)	25 (13.0%)	0.85
DA	3 (3.6%)	5 (12.2%)	3 (4.5%)	11 (5.7%)	
DMG	22 (26.2%)	8 (19.5%)	17 (25.4%)	47 (24.5%)	
GBM	40 (47.6%)	21 (51.2%)	29 (43.3%)	90 (46.9%)	
Others^a^	10 (11.9%)	2 (4.9%)	7 (10.4%)	19 (9.9%)	
preoperative HCP						
No	50 (59.5%)	32 (78.0%)	53 (79.1%)	135 (70.3%)	0.039^*^
Yes	34 (40.5%)	9 (22.0%)	14 (20.9%)	57 (29.7%)	
postoperative HCP						
No	63 (75.0%)	37 (90.2%)	60 (89.6%)	160 (83.3%)	0.058
Yes	21 (25.0%)	4 (9.8%)	7 (10.4%)	32 (16.7%)	
Preoperative KPS						
Mean (SD)	72.6 (16.9)	74.4 (15.3)	74.0 (16.1)	73.5 (16.2)	0.943
Median [Min, Max]	80.0 [20.0, 90.0]	80.0 [20.0, 90.0]	80.0 [20.0, 90.0]	80.0 [20.0, 90.0]	
Postoperative KPS						
Mean (SD)	45.5 (20.0)	58.3 (15.0)	51.2 (14.5)	50.2 (17.8)	0.002^**^
Median [Min, Max]	50.0 [0, 80.0]	60.0 [20.0, 80.0]	50.0 [30.0, 80.0]	50.0 [0, 80.0]	
H3K27M mutant						
No	69 (82.1%)	35 (85.4%)	52 (77.6%)	156 (81.3%)	0.781
Yes	15 (17.9%)	6 (14.6%)	15 (22.4%)	36 (18.8%)	
IDH mutant						
No	81 (96.4%)	40 (97.6%)	63 (94.0%)	184 (95.8%)	0.819
Yes	3 (3.6%)	1 (2.4%)	4 (6.0%)	8 (4.2%)	
G_1p19q codeletion						
No	84 (100%)	41 (100%)	63 (94.0%)	188 (97.9%)	0.055
Yes	0 (0%)	0 (0%)	4 (6.0%)	4 (2.1%)	
Time						
Mean (SD)	7.94 (14.9)	14.6 (25.3)	21.1 (18.7)	14.0 (19.6)	<0.001^***^
Median [Min, Max]	3.50 [1.00, 108]	7.00 [1.00, 159]	14.0 [1.00, 81.0]	8.50 [1.00, 159]	

SR/SC, surgery + radiotherapy/chemotherapy; SRC, surgery + radiochemotherapy; AA, anaplastic astrocytoma; DA, diffuse astrocytoma; DMG, diffuse midline glioma; GBM, glioblastoma; HCP, hydrocephalus; KPS, Karnofsky Performance Scale score; OS, overall survival. ^a^Due to the long time span of the data collected in this study, during which the WHO was updated, the no longer used pathological classifications were grouped into others;

***, p< 0.001; **, p<0.01; *, p<0.05

### Kaplan-Meier survival analysis

**Figure 2:**
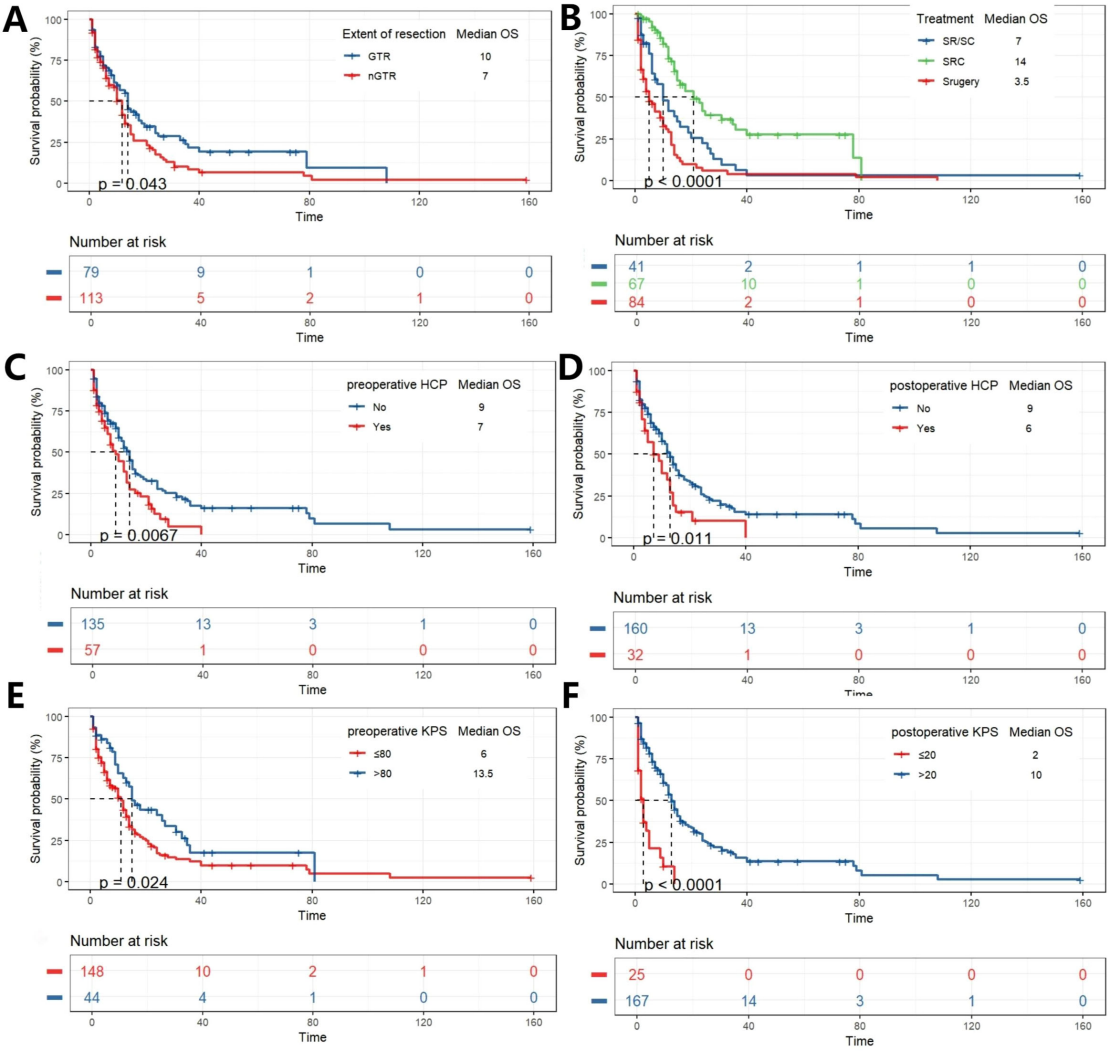
K-M analysis determining the impact of variables on OS (months). Stratified by EOR **(A)**, Treatment **(B)**, Preoperative HCP **(C)**, postoperative HCP **(D)**, preoperative KPS **(E)**, and postoperative KPS **(F)**.

K-M curve is drawn to analyze the influence of variables on OS. The preoperative and postoperative KPS scores were classified according to the optimal cut-off value. Gross total resection, surgery combined with chemoradiotherapy, absence of hydrocephalus before and after surgery, preoperative KPS scores >80, and postoperative KPS scores >20 were associated with better survival outcomes (**Figure 2**). The K-M survival analysis revealed a significant difference in overall survival among the three treatment groups (p < 0.0001). *Post-hoc* pairwise comparisons with Bonferroni correction for multiple testing were subsequently performed. The adjusted analyses demonstrated that patients in the SRC group had significantly longer overall survival compared to those in the Surgery alone group (p = 5.2 × 10^-^¹^0^) and the SR/SC group (p = 0.002). A significant survival benefit was also observed in the SR/SC group compared to the Surgery alone group (p = 0.036) (**Supplementary Figure 1**).

**Figure 3:**
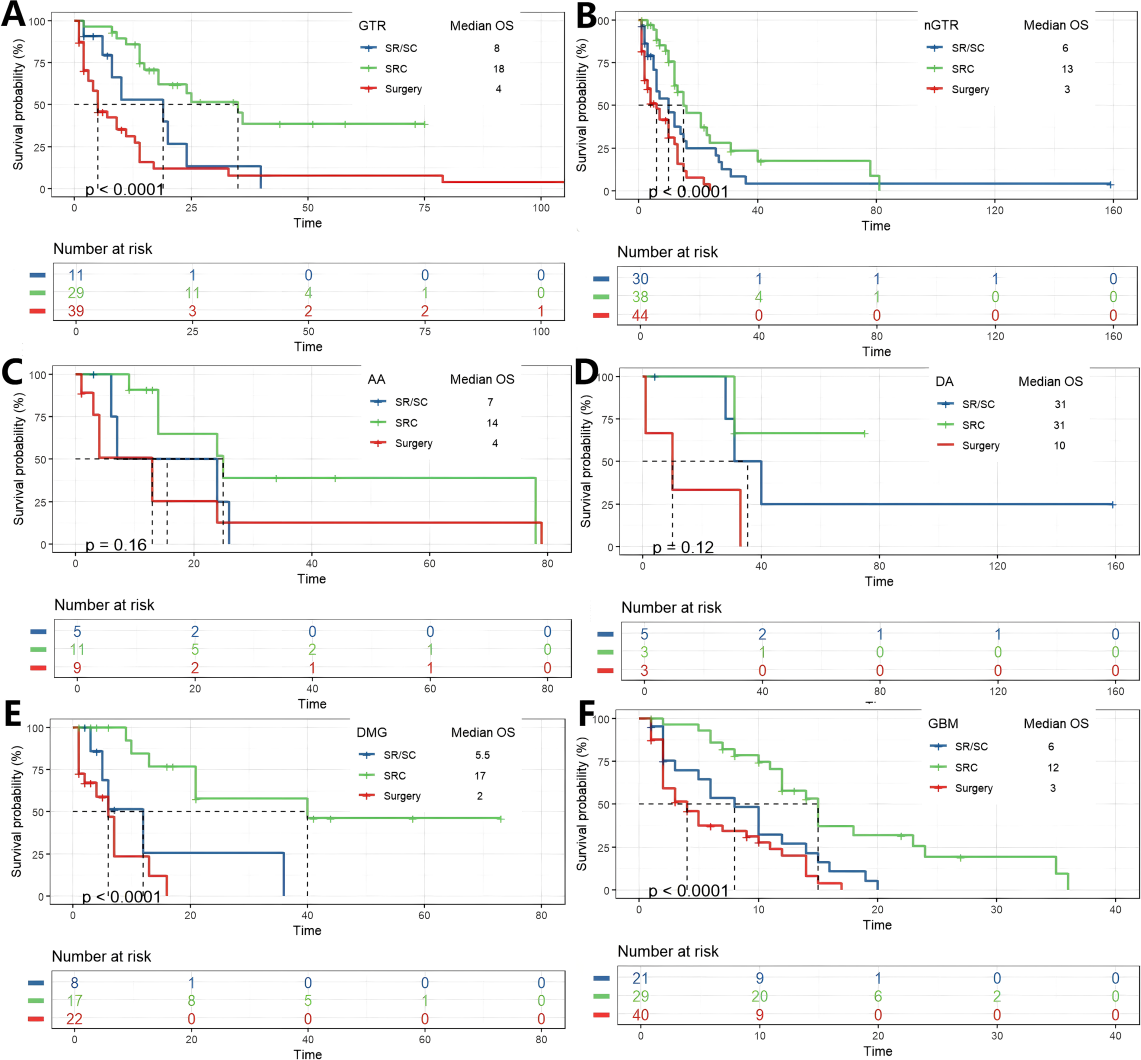
K-M analysis determining the impact of different treatments on OS(months) in subgroups. Grouped by EOR **(A, B)**, and diagnosis of AA, DA, DMG and GBM.**(C-F)**.

Subgroup analysis was conducted based on the degree of tumor resection, whether the patient had hydrocephalus before and after surgery, and the pathological diagnosis of the tumor. They were divided into two subgroups (nGTR, GTR) according to the degree of tumor resection. Among patients who achieved GTR, K-M analysis revealed a significant overall difference in survival between the three treatment strategies (p < 0.0001). *Post-hoc* pairwise comparisons with Bonferroni adjustment identified the source of this difference: patients receiving SRC had a significantly longer overall survival compared to those who underwent surgery alone (p = 8.6 × 10^-6^). The survival advantage of SRC over SR/SC approached statistical significance (p = 0.042). In contrast, no significant survival difference was observed between the SR/SC and Surgery alone groups (p = 0.559). In patients nGTR, a significant disparity in overall survival was observed across the treatment groups (p < 0.0001). *Post-hoc* pairwise analyses with Bonferroni correction delineated a hierarchy of efficacy: the SRC regimen demonstrated a profound survival advantage over surgery alone (p = 8.7 × 10^-6^). Similarly, the SR/SC regimen was associated with a statistically significant improvement in survival compared to surgery alone (p = 0.044). However, no statistically significant difference in survival was detected between the SRC and SR/SC groups (p = 0.151). Subgroup analyses were conducted for diagnosis of AA, DA, DMG and GBM in this study, K-M analysis revealed a significant overall difference in survival between the three treatment strategies in DMG and GBM subgroups (p<0.0001), while the statistical differences were not significant in the AA and DA subgroups. *Post hoc* pairwise analysis corrected by Bonferroni showed a similar trend in patients diagnosed with DMG and GBM, that is, patients who received SRC had a significantly longer overall survival compared with those who only received surgery (p = 3.4×10– (5) and p = 7.3×10 ^-6^, respectively). There was a statistically significant difference in the survival advantage of SRC compared with SR/SC (p = 0.013 and p = 0.005, respectively). In contrast, no significant survival difference was observed between the SR/SC group and the surgery alone group (p = 0.632, p = 0.326, respectively) (**Figure 3**; **Supplementary Figure 2**).

**Figure 4:**
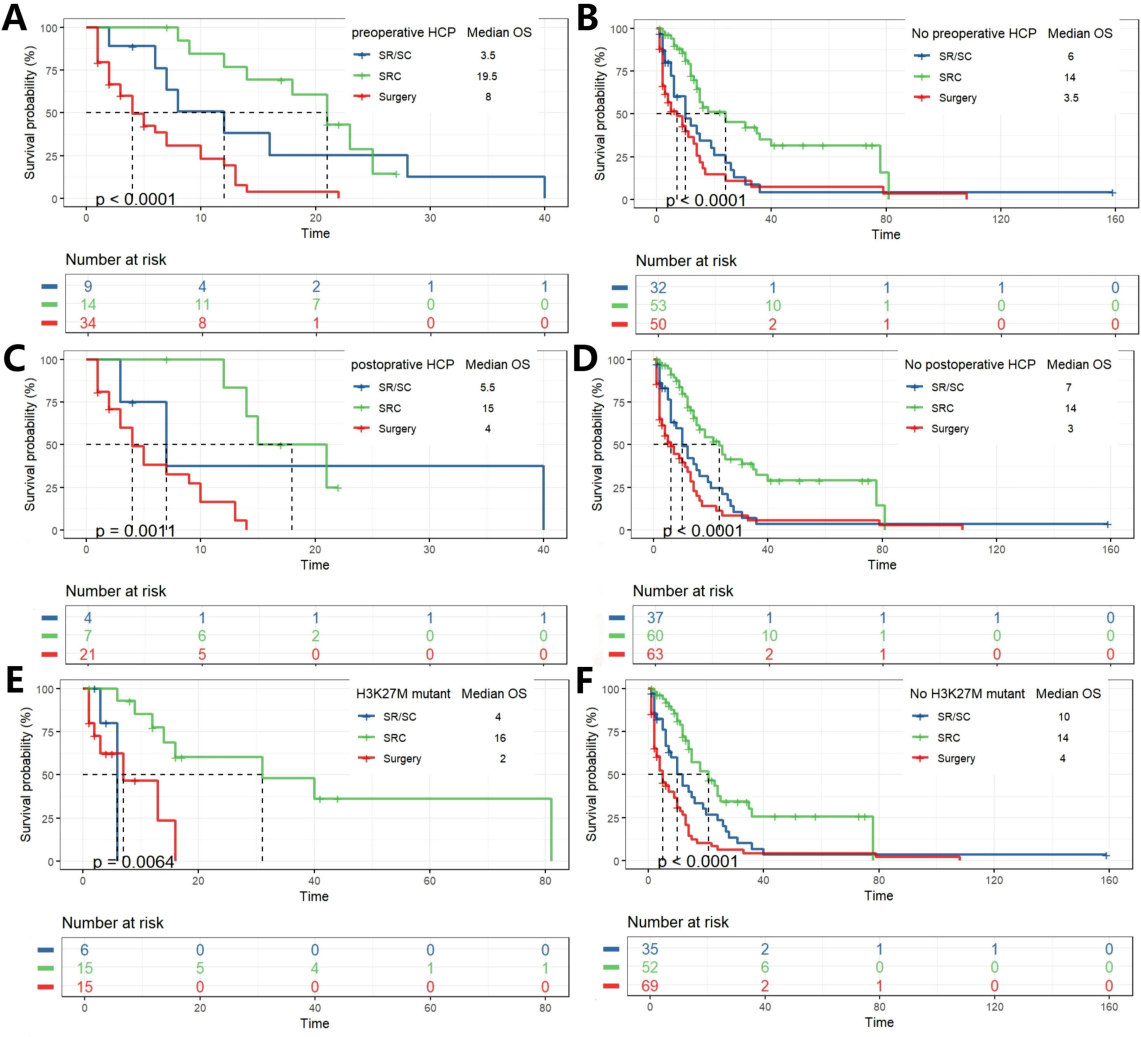
K-M analysis determining the impact of different treatments on OS(months) in subgroups. Grouped by with and without hydrocephalus pre- and postoperation **(A–D)**. Grouped by with and without H3K27M mutation **(E, F)**.

The impact of treatment modality on survival was further analyzed in subgroups stratified by hydrocephalus status. Among patients with preoperative hydrocephalus, a significant overall survival difference was observed (p < 0.0001). Bonferroni-adjusted pairwise comparisons revealed that the SRC regimen conferred a profoundly significant survival advantage over surgery alone (p = 1.5 × 10^-5^). No other pairwise comparisons reached statistical significance after adjustment. A nearly identical pattern was observed in patients with postoperative hydrocephalus (p = 0.0011). Again, after correction for multiple testing, the survival benefit of SRC over surgery alone remained overwhelmingly significant (p = 0.00059), while no other comparisons were statistically significant. A distinct pattern emerged in patients without hydrocephalus. The overall survival difference was highly significant (p < 0.0001 for both pre- and post-operative subgroups). After Bonferroni adjustment, pairwise comparisons not only confirmed the profound survival advantage of SRC over surgery alone (p = 5.4 × 10^-5^ and p = 1.3 × 10^-6^, respectively) but also revealed a statistically significant superiority of SRC over the SR/SC regimen (p = 0.007 and p = 0.003, respectively). Stratification by H3K27M mutation status revealed distinct response patterns to therapy. In the H3K27M-mutant cohort, survival differed significantly between groups (p = 0.0064). *Post-hoc* analysis demonstrated that only the SRC regimen provided a significant survival benefit over surgery alone (p = 0.009) and was superior to the SR/SC regimen (p = 0.012). The SR/SC regimen did not confer a significant survival advantage compared to surgery alone (p = 1.0). In contrast, within the H3K27M-wildtype cohort (p < 0.0001), a clear gradient of efficacy was observed. All treatment modalities outperformed surgery alone (SRC vs. Surgery, p = 6 × 10^-8^; SR/SC vs. Surgery, p = 0.045), and the SRC regimen was significantly superior to SR/SC (p = 0.015) (**Figure 4**; **Supplementary Figure 3**).

### Lasso-Cox regression analysis and comparison of different models

**Figure 5:**
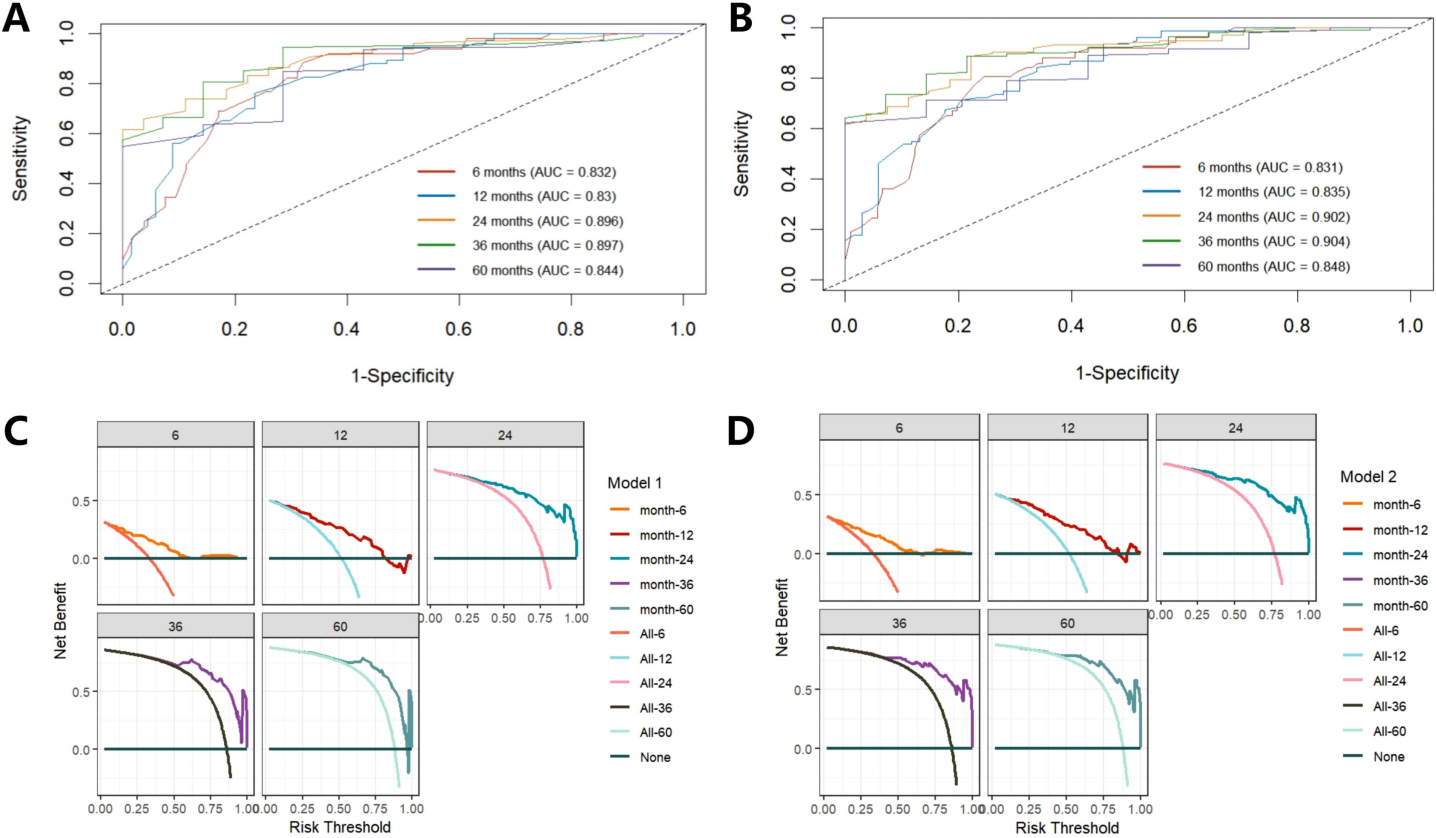
Comparison of time-ROC curve and DCA curve between Model 1 and Model 2. Time-ROC of Model 1 shows a good classification performance **(A)**, while Model 2 is better than Model 1 **(B)**. DCA curve shows Model 2 has a higher clinical application value than Model 1 **(C, D)**.

**Figure 6:**
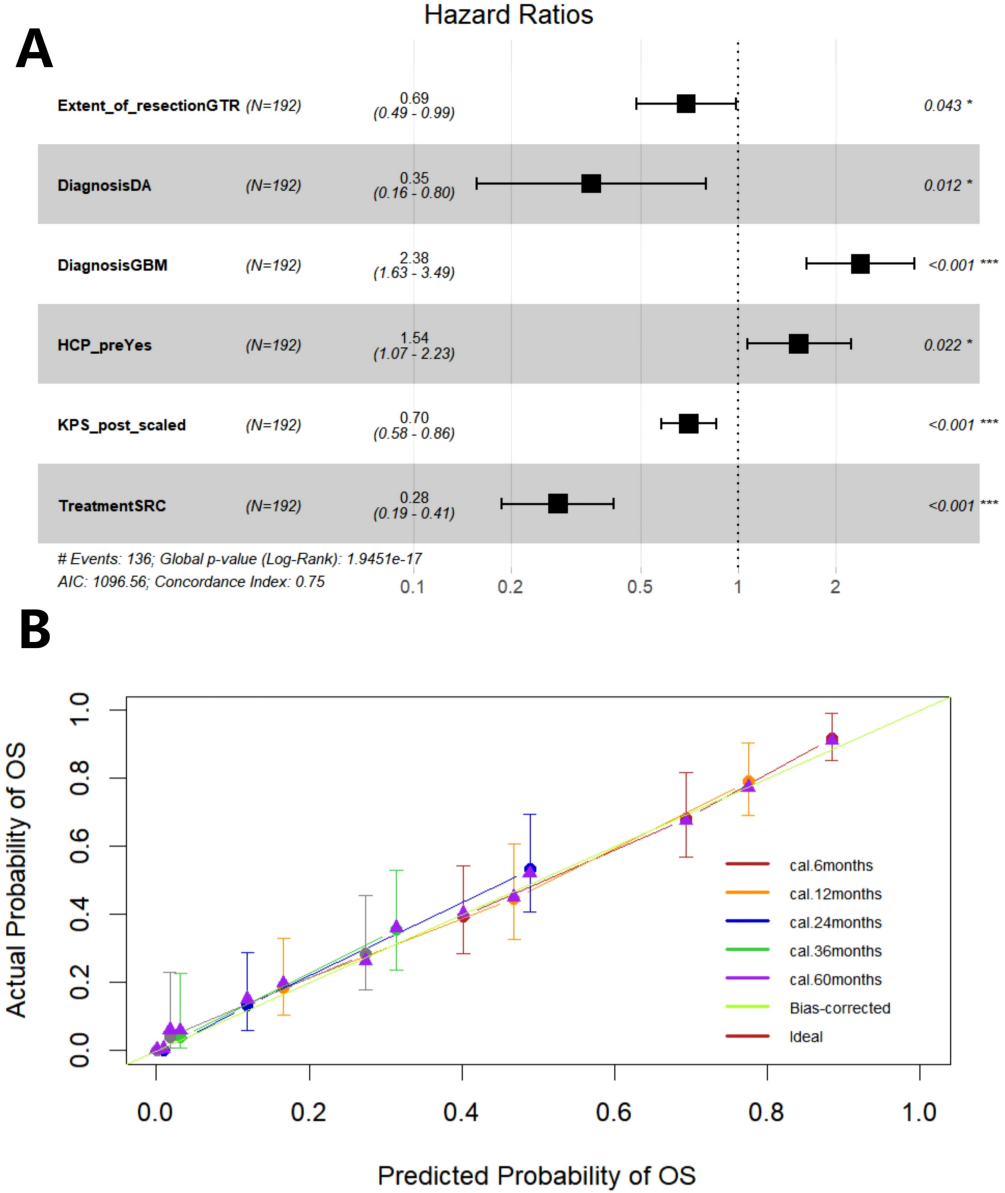
The forest plot shows the hazard ratio of the variable and the 95% confidence interval **(A)**. Calibration curve of Model 2 **(B)**. GTR, gross total resection; DA, diffuse astrocytoma; GBM, glioblastoma; HCP, hydrocephalus; KPS, Karnofsky Performance Scale score; SRC, surgery + radiochemotherapy.

Firstly, through lasso regression analysis, we select the optimal lambda (lambda.1se) to screen variables in order to fit the model with low complexity, high stability and better generalization ability. Lasso regression analysis screened out four characteristic variables, including diagnosis, treatment, preoperative HCP and postoperative KPS. Further, these four variables were included in cox regression to establish Model 1. Multivariate COX regression analysis showed that these four variables were all independent prognostic factors for OS (p<0.05). Considering the clinical importance of EOR, based on the principle of “clinically informed modeling”, we forcibly added the EOR variable on the basis of Model 1 to further construct the prediction Model 2. Then we calculated the C-index, AIC and BIC of the two models respectively. The results are shown in **Table 2**. The C-index of the two models is similar (C-index = 0.752 vs. 0.751), the prediction performance of OS is comparable. However, the AIC of Model 2 is lower (ΔAIC = -2.201), the BIC is close (ΔBIC = 0.712 << 6), indicating that Model 2 has a better goodness of fit, while adding the EOR variable has no significant effect on the complexity of the model. In addition, the time-ROC curves and AUC of Model 1 and Model 2 were compared. The predictive performance of model 2 for OS at 6-, 12-, 24-, 36-, and 60 months was all superior to that of Model 1 (**Figures 5A, B**). The DCA curve was plotted to compare the clinical application value of the two models. Model 2 outperformed Model 1 in both short-term and long-term clinical net benefits, especially having strong clinical application value in predicting long-term outcomes (**Figures 5C, D**). Therefore, we choose Model 2 with better prediction efficiency as the final prediction model. Factors associated with better survival outcomes included GTR, high postoperative KPS score, diagnosis of DA, SRC (**Figure 6A**).

**Table 2:** Predictive performance comparison between model 1 and model 2.

LASSO-COX model	Variables	C-index	AIC	ΔAIC	BIC	ΔBIC
Model 1	4	0.751 (95% CI:0.710-0.792)	1098.759	-2.201	1113.322	0.712
Model 2	5	0.752 (95% CI:0.720-0.793)	1096.558		1114.034	

Then, through 200 times internal validations of bootstrap, the C-index after model correction was 0.742 (95% CI: 0.716-0.768). The optimism degree was 0.009, which was much lower than the warning value of 0.05, indicating a slight degree of overfitting of the model, the final model(Model 2) had both good generalization ability and stability. The calibration curve of the corrected model was plotted (**Figure 6B**). It can be seen that the prediction curves at each time point are close to the diagonal (especially in the long-term outcome), that is, the prediction probability of the prediction model is close to the actual probability. The model has good accuracy in predicting prognosis, especially the long-term prognosis.

### Construction of prognostic nomogram and risk stratification K-M analysis

**Figure 7:**
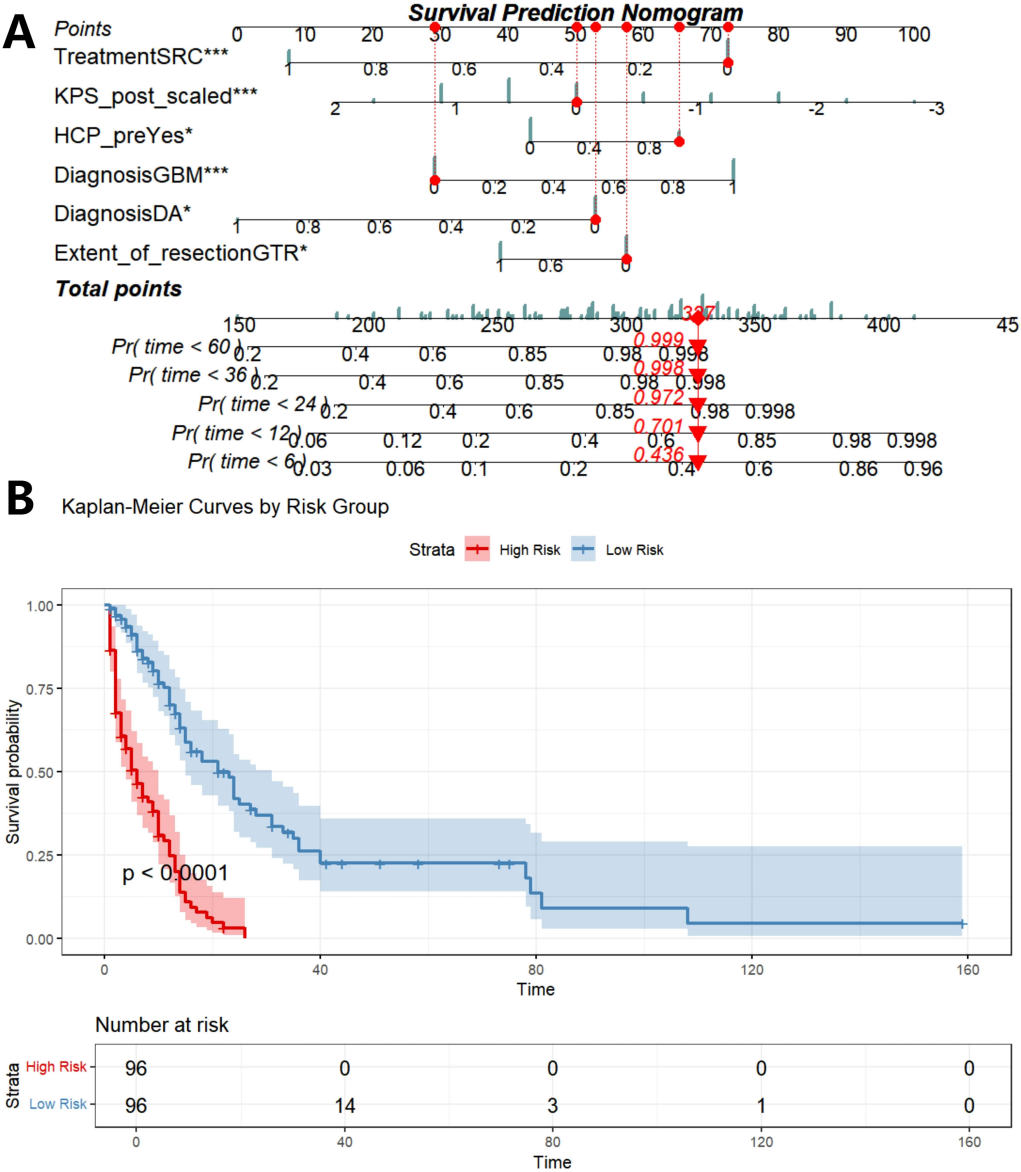
Construction of prognostic nomogram based on five independent prognostic factors **(A)**, K-M analysis determined the impact of risk stratification on OS based on prognostic nomogram **(B)**. SRC, surgery combined with chemoradiotherapy; SR/SC, surgery combined with radiotherapy or chemotherapy; KPS_post, postoperative KPS score; HCP_pre, preoperative HCP; DA, diffuse astrocytoma; AA, anaplatic astrocytoma; DMG, diffuse midline glioma; GBM, glioblastoma; Pr, probability.

Five prognostic factors (EOR, diagnosis, preoperative HCP, preoperative KPS, treatment) are incorporated to construct prognostic columns of OS at 6-, 12-, 24-, 36-, and 60 months (**Figure 7A**). The total score of each patient was calculated and divided into two death risk subgroups, high risk group and low risk group, according to the median total score of the nomogram. The K-M curve was drawn to show that there was a statistically significant difference in OS between the two groups (P<0.001), indicating that the prognostic column had good clinical value (**Figure 7B**).

### Analysis of the prognostic value and statistical power of molecular markers

The distribution of key molecular markers and the results of their prognostic analyses in the study cohort are summarized in **Table 3**. The positivity rate for H3K27M mutation was 18.8% (36/192), with 18 events (50%) occurring in the positive group. Univariable survival analysis indicated that H3K27M mutation was associated with poorer prognosis, although the difference did not reach statistical significance (HR = 0.71, 95% CI: 0.43–1.17, p = 0.184). The IDH mutation had a low positivity rate (4.2%, 8/192), with only one event observed in this subgroup. Its univariable analysis suggested a potential trend toward better prognosis, albeit with an extremely wide confidence interval (HR = 0.15, 95% CI: 0.02–1.06, p = 0.057). The 1p/19q codeletion showed the lowest positivity rate (2.1%, 4/192), and no events were observed in the positive group, precluding a reliable estimation of the hazard ratio (HR ≈ 0, p = 0.994).

**Table 3:** Overview and statistical efficacy analysis of molecular markers.

Molecular biomarker	Positive cases	Events in positive group	Events in negative group	HR (95% CI)	P value	Statistical power	Required sample size(80% Power)
H3K27M mutation	36	18	118	0.71 (0.43-1.17)	0.184	0.646	NA
IDH mutation	8	1	135	0.15 (0.02-1.06)	0.057	0.877	NA
1p19q co-deletion	4	0	136	0.00 (0.000-Inf) ^1^	0.994^1^	NA^1^	665(Total)^2^

^1^No events in positive group, unable to calculate. ^2^Based on the assumption HR = 0.3 and the current percentage of positive cases (2.1%).

Statistical power analysis indicated that the study was underpowered to detect prognostic effects for these molecular markers. Under the current sample size and event distribution, the power to detect an effect for H3K27M mutation was 64.6%, and for IDH mutation, 87.7%. Although the estimated power was relatively high for IDH mutation, this result remains highly uncertain due to the single event in the positive subgroup. For 1p/19q codeletion, a cohort of approximately 665 patients would be required to reliably detect a hypothesized effect (HR = 0.3), which is 3.5 times the current sample size (**Table 3**).

## Discussion

This study is the first to analyze the effects of surgical resection combined with postoperative chemoradiotherapy on the survival outcomes of patients with thalamic glioma. The surgical treatment of thalamic gliomas is extremely challenging due to the specificity of the thalamic anatomic location, and despite advances in the surgical treatment of thalamic gliomas due to the development of microneurosurgery, the results are still unsatisfactory (22, 23). In this paper, the effects of systematic treatment of surgery combined with postoperative chemoradiotherapy on the survival of patients with thalamic glioma were explored, and a prognostic model of thalamic glioma was established.

Based on survival curve analysis, we found that the degree of surgical resection significantly affected survival outcomes, and GTR was associated with better survival outcomes, which is consistent with our previous findings (18). At present, there is no unified treatment plan for thalamic glioma in clinical practice, especially the roles of radiotherapy and chemotherapy in it are highly controversial. Some studies have reported that the survival outcome of patients with thalamic glioma after adjuvant therapy is worse (4), while other research results have shown the positive effects of radiotherapy and chemotherapy (15, 24, 25). Our previous studies have also reported that postoperative adjuvant therapy helps to prolong overall OS, even though postoperative adjuvant therapy was not an independent prognostic factor for thalamic glioma (18). In this study, we included a larger sample size. The results showed that postoperative chemoradiotherapy, as an independent prognostic factor, could improve the prognosis of thalamic glioma, and the median OS was significantly prolonged.

We also employed multi-level subgroup analyses to evaluate therapeutic heterogeneity in adult thalamic glioma. Across all subgroups—including those stratified by EOR, HCP status, or molecular profile—survival outcomes were significantly better with the SRC regimen than with surgery alone. This consistent benefit strongly supports SRC as a cornerstone treatment for this population. In subgroups with more favorable prognosis (e.g., without HCP or H3K27M mutation), a clear efficacy gradient of SRC > SR/SC > Surgery was observed, indicating that treatment intensity correlates positively with survival in patients with better clinical status. While SRC is optimal, SR/SC represents a valid alternative. By contrast, within poor-prognosis subgroups (e.g., H3K27M mutant tumors), a different pattern of which SRC remained effective, whereas SR/SC showed no significant survival benefit over surgery alone emerged. This suggests an “intensity threshold” for treating highly aggressive tumors; only above this threshold—achieved with full multimodal SRC—can meaningful survival benefits be realized. Notably, the thalamus is a midline structure (26), and H3K27M-mutant diffuse midline glioma is classified by WHO as grade IV with typically poor prognosis (27–31). Although limited sample size restricted formal prognostic analysis of H3K27M status, subgroup analyses consistently showed that SRC improved survival irrespective of mutation status, while outcomes remained worse in H3K27M-mutant cases compared to wild-type—consistent with existing literature (32–34). Moreover, H3K27M status helped predict response to treatment intensity. Despite these heterogeneities, maximized resection and standardized systemic therapy (SRC) should be strongly recommended as the standard treatment regimen for adult thalamic glioma.

To further explore the factors influencing the survival outcome of thalamic glioma, we conducted LASSO regression analysis. Although the data-driven approach (Model 1) did not select EOR, this might be due to the collinearity between EOR and Treatment, while the LASSO algorithm tends to focus on more important variables or is limited by the smaller sample size, led to the incorrect elimination of EOR, yet its core role in influencing the prognosis of glioma has been confirmed by multiple studies (35–37). Considering the ultimate clinical practicability of the model, based on the “clinically informed modeling”, we included EOR variables to construct the Model 2. This method emerged in multiple classic cases (such as Framingham Heart Study Risk Score) (38), expanding the clinical applicability of the model. Considering the influence of subjectively adding variables on the model’s performance, we compared two models. Results showed that adding the EOR variable improved the predictive performance and clinical application value of the model. Moreover, 200 bootstrap samplings provided reliable internal verification. The testing process of the “new dataset” was simulated through resampling, compensating for the limitation of this study that the sample size was small and the dataset could not be split. The verification results show that the final model has strong generalization ability and high stability.

Interestingly, in the time-ROC curve, calibration curve and DCA curve of the final model, it was shown that the model predicted the long-term outcome (time > 24 months) accurately and had strong clinical application value. However, it had limitations in predicting the short-term outcome (6 months). On the one hand, this might be because the occurrence of short-term events was affected by more acute and random factors, which is difficult to predict. On the other hand, it might be because the important factors included (such as chemoradiotherapy in the treatment methods) has not yet fully demonstrated its impact in the short term. Further expanding the sample size and incorporating more acute physiological indicators and early treatment response indicators may improve the short-term predictive performance of the model.

This study has several important limitations that should be considered when interpreting the results. First, its single-center, retrospective design inherently influences case selection, surgical practices, and postoperative care, potentially limiting the generalizability of our findings to other institutions with different protocols and patient populations. Second, the prolonged inclusion period (2009–2024) introduces temporal heterogeneity, as treatment standards and supportive care evolved substantially over this timeframe. Although we aimed for standardized protocols, these unmeasured temporal trends could influence long-term outcomes. A central challenge stems from the evolution of CNS tumor classification. Diagnoses were based on contemporary WHO criteria at the time, not a uniform reclassification per WHO 2021. This introduces spectrum bias, as historical groups like astrocytoma or oligodendroglioma likely encompass molecularly distinct entities by current standards (e.g., IDH-mutant and IDH-wildtype tumors), which may have diluted the prognostic signal of pathological diagnosis in our model. Consequently, exploratory analyses of molecular markers (IDH mutation, 1p/19q codeletion, H3K27M) were severely limited by their low retrospective availability and prevalence. The positivity rates were exceedingly low, and the number of events in positive subgroups was negligible. Therefore, any statistical findings related to these markers are essentially uninterpretable due to profound imprecision (as seen in implausibly wide confidence intervals); we explicitly caution against any overinterpretation and emphasize these analyses are strictly exploratory. Finally, our study focused on survival and traditional clinical metrics. We did not assess functional or patient-reported quality-of-life (QOL) outcomes, which are critical for evaluating the holistic benefit of treatment strategies, especially in weighing survival gains against treatment-related toxicities. Despite these limitations, the identified clinical prognostic factors demonstrate robust predictive value. Our model provides a validated prognostic framework based on readily available clinical data. Future prospective, multi-center studies with uniform molecular classification per contemporary standards, integrated with standardized QOL metrics, are essential to refine personalized prognostic models.

## Conclusions

This study conducted survival analysis on adult thalamic gliomas treated with three different regimens(surgery only; SR/SC; SRC) and established a survival prognosis model based on LASSO-COX regression analysis. It was found that the standardized and systematic treatment of maximum resection within the safe range + chemoradiotherapy could significantly prolong the overall OS of patients with thalamic glioma, which was instructive for their clinical treatment. The model has strong clinical application value in predicting long-term outcomes.

## Data Availability

The datasets presented in this article are not readily available because the author has no permission to share the dataset. Requests to access the datasets should be directed to yangyuan@wchscu.cn.

## Supplementary material

Supplementary Figure 1 K-M analysis of Boferroni multicorrected p values determining the impact of treatment on OS (months).

Supplementary Figure 2 K-M analysis of Boferroni multicorrected p values determining the impact of different treatments on OS(months) in subgroups. Grouped by EOR **(A, B)**, and diagnosis of DMG and GBM. **(C, D)**.

Supplementary Figure 3 K-M analysis of Boferroni multicorrected p values determining the impact of different treatments on OS(months) in subgroups. Grouped by with and without hydrocephalus pre- and postoperation **(A–D)**. Grouped by with and without H3K27M mutation **(E, F).**
