# Supplementary figures and images for: The impact of maximized resection and standardized systemic therapy on overall survival in adult patients with thalamic gliomas
Source: Front Oncol. 2025 Oct 30;15:1681695. doi: 10.3389/fonc.2025.1681695 (PMC12611655; doi:10.3389/fonc.2025.1681695)

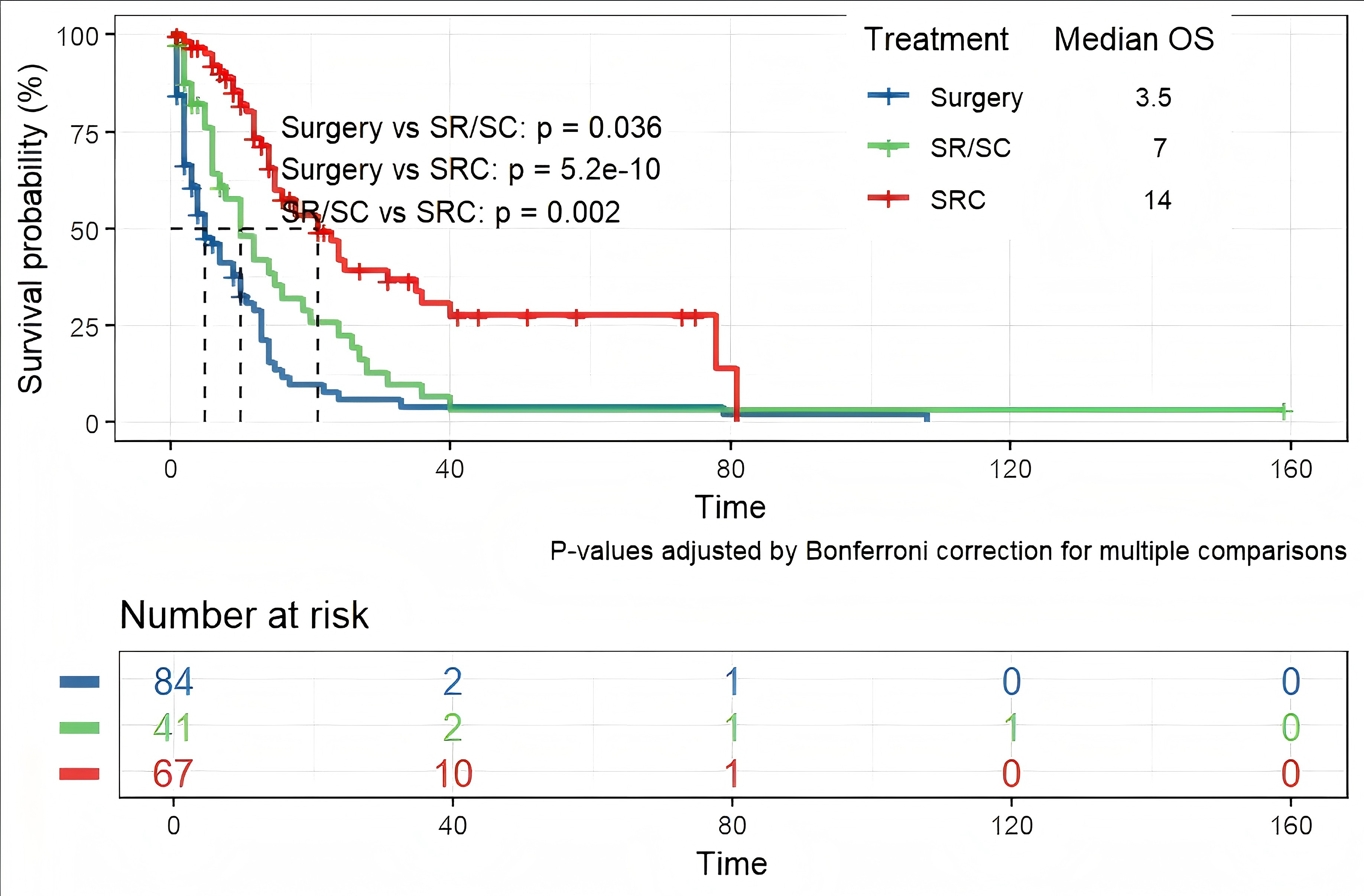

Supplement: Supplementary Figure 1 — K-M analysis of Boferroni multicorrected p values determining the impact of treatment on OS (months). [file Image1.jpeg]

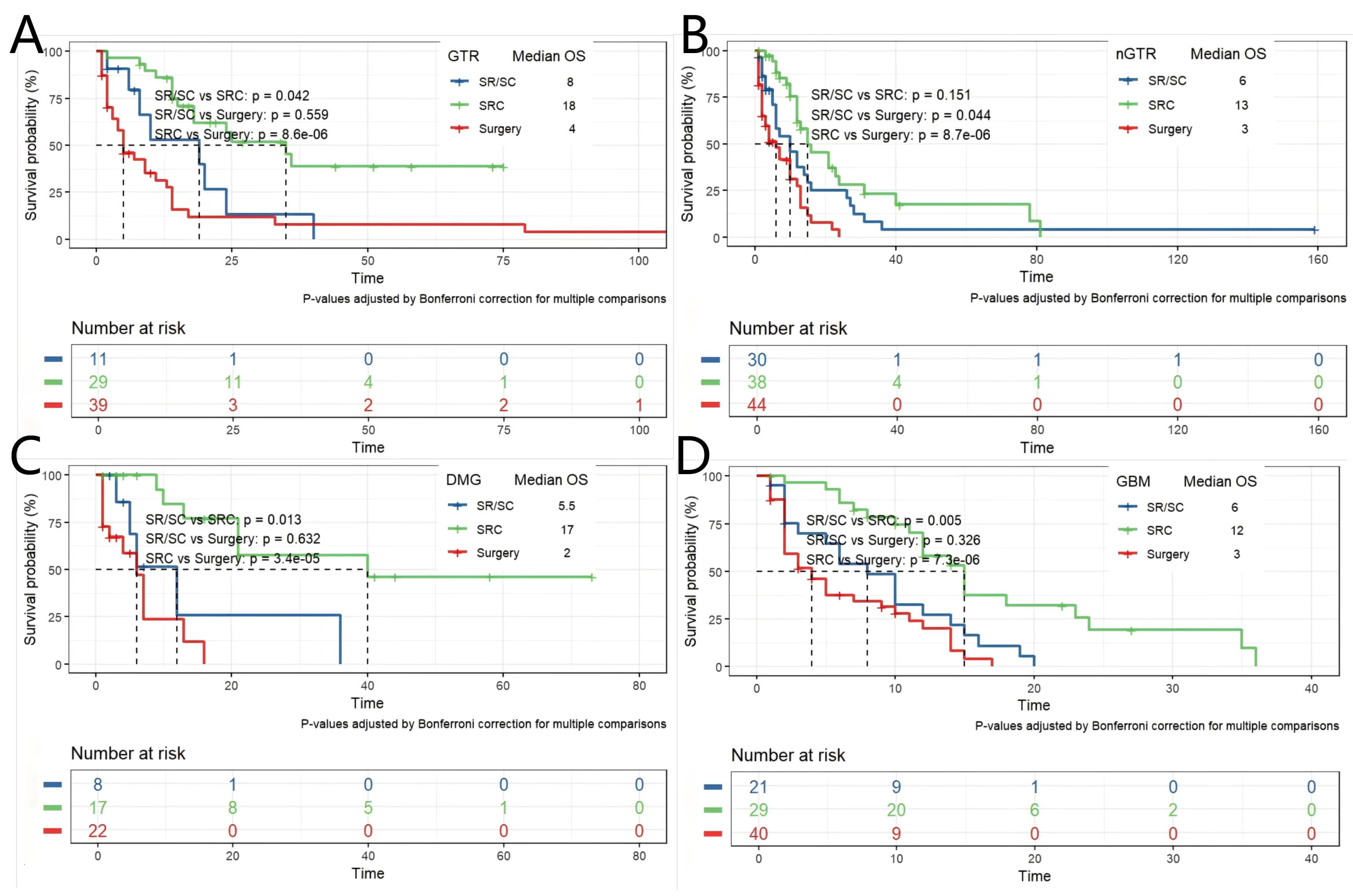

Supplement: Supplementary Figure 2 — K-M analysis of Boferroni multicorrected p values determining the impact of different treatments on OS(months) in subgroups. Grouped by EOR (A, B), and diagnosis of DMG and GBM. (C, D). [file Image2.jpeg]

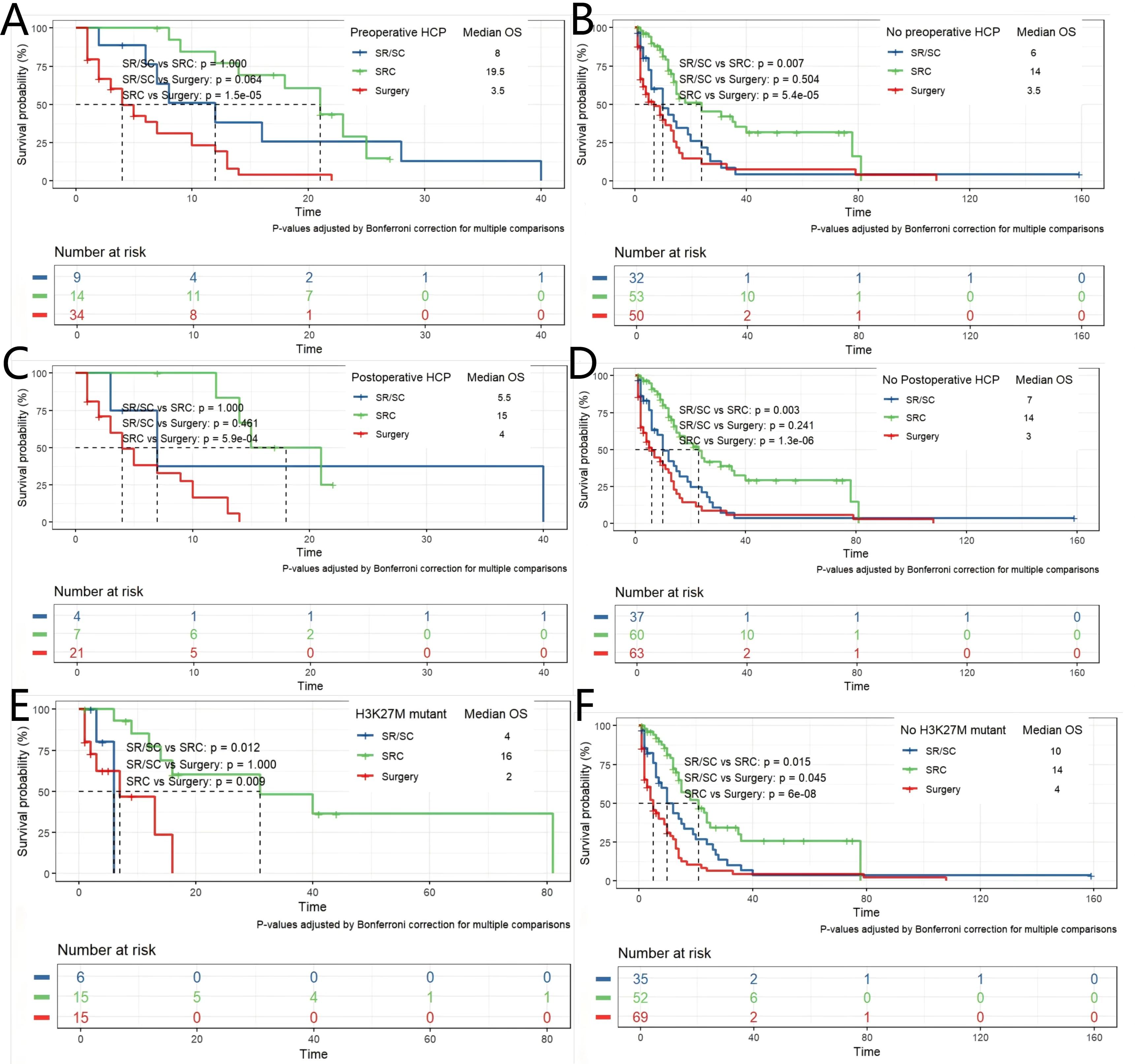

Supplement: Supplementary Figure 3 — K-M analysis of Boferroni multicorrected p values determining the impact of different treatments on OS(months) in subgroups. Grouped by with and without hydrocephalus pre- and postoperation (A–D). Grouped by with and without H3K27M mutation (E, F). [file Image3.jpeg]
